# An Observational Study of Dialogue about Uncertainty in Clinician-Family Counseling Conversations Following Prenatal Diagnosis of Complex Congenital Heart Disease

**DOI:** 10.1016/j.pecinn.2024.100265

**Published:** 2024-02-13

**Authors:** Kelly W. Harris, Kelsey Schweiberger, Ann Kavanaugh-McHugh, Robert M. Arnold, Jessica Merlin, Judy C. Chang, Nadine A. Kasparian

**Affiliations:** a Department of Pediatrics, University of Pittsburgh School of Medicine, Pittsburgh, PA, USA; b Department of Pediatrics, Vanderbilt University Medical Center, Nashville, TN, USA; c Department of General Internal Medicine, University of Pittsburgh School of Medicine, Pittsburgh, PA, USA; d Department of Obstetrics, Gynecology, and Reproductive Sciences, University of Pittsburgh School of Medicine, Pittsburgh, PA, USA; e Department of Pediatrics, University of Cincinnati College of Medicine, Cincinnati, OH, USA

**Keywords:** Communication, Uncertainty, Parent experience, Fetal, Diagnosis, Congenital heart disease

## Abstract

**Objective:**

Families who receive a prenatal diagnosis of complex congenital heart disease (cCHD) often experience severe psychological distress and identify uncertainty as a key source of that distress. This study examined clinician-family conversations during initial fetal cardiology consultations to identify the topics of uncertainty discussed.

**Methods:**

In this observational, qualitative study, initial fetal cardiology consultations were audio-recorded, transcribed verbatim, and coded by two independent coders. A codebook was inductively and deductively developed and applied. This content analysis focused on uncertainty-related codes and associated themes.

**Results:**

During 19 consultations including five clinicians, 13 different cardiac diagnoses were discussed (seven with high mortality risk). Median consultation length was 37 min (IQR: 26–51), with only 11% of words spoken by families. On average, 51% of total words spoken focused on uncertainty in relation to cardiac diagnosis, etiology, comorbidities, prognosis, childbirth, therapeutics, and logistics. Family-initiated discussion on uncertainty largely focused on childbirth and pregnancy and postpartum logistics.

**Conclusions:**

Half of dialogue within initial fetal cardiology encounters discussed uncertainty surrounding prenatally diagnosed cCHD. Parent and clinician perspectives should be gathered on the essential content and optimal delivery of uncertainty-related topics.

**Innovation:**

This study is conceptually and methodologically innovative as one of the first to examine audio-recorded dialogue between fetal cardiology clinicians and families.

## Highlights

• Half of dialogue in fetal cardiology consultations discusses uncertainty.
• Clinicians introduce and discuss most topics relating to uncertainty.
• Clinicians focus mostly on cardiac diagnosis, prognosis, and therapeutics.
• Parents most commonly ask about childbirth and logistics.

## 1 Introduction

Congenital heart disease (CHD) occurs in nearly 1% of infants in the United States, making it the most common birth defect. [[1], [2], [3]] Approximately one-quarter of infants with CHD have life-threatening symptoms requiring intensive care unit (ICU) admission and invasive intervention. [4] Diagnoses of complex CHD (cCHD) are frequently made prenatally, which may improve clinical management and outcomes for some infants [5] but for parents, often elicits severe psychological distress. [6,7] Compared with those who learn about their child's heart condition after birth, parents who receive a prenatal diagnosis report greater anxiety and depressive symptoms, lower life satisfaction, and greater suicidal ideation. [8,9] High levels of psychological distress can adversely affect parent and family health and functioning [10] as well as child developmental outcomes. [[11], [12], [13]]

In our prior qualitative work, we found that after receiving a prenatal cardiac diagnosis, parents and other family members identified uncertainty as a key source of their distress, [14] consistent with prior research on CHD [15] and other potentially life-limiting conditions. [7,14,[16], [17], [18], [19], [20], [21], [22], [23], [24], [25], [26]] Families described areas of uncertainty related to short- and longer-term healthcare logistics, as well as uncertainties inherent to a prenatal cCHD diagnosis including diagnosis itself, prognosis, and potential interventions due to evolving anatomy and physiology, and limitations of technology. [14] Parental uncertainties include these clinical topics but also extend to also include psychological, social, financial, familial, and logistical aspects.

As Han et al. (2011) describes, uncertainty occurs when something is “not clearly identified or defined” or there is “lack of knowledge about some aspect of reality.” [27] When an individual faces uncertainties related to a diagnosed or undiagnosed medical condition, they are experiencing *illness uncertainty*. [28] Illness uncertainty is a multidimensional experience, incorporating the cause, topic, and individual perspective of uncertainty. In this study, we describe the causes of uncertainty as domains – called dimensions by Mishel (1990), sources by Han et al. (2011), and meanings by Babrow (1998); domains include unpredictability (i.e., that future outcomes are indeterminate or unknown), complexity (i.e., complex or complicated features of information that may limit parental understanding), and lack of information (i.e., lack of reliable or adequate information given to parents). [18,[27], [28], [29], [30], [31], [32]]

Despite our knowledge of parental uncertainties after a prenatal cCHD diagnosis, little is known about how clinicians and families discuss uncertainty during the first fetal cardiology consultation encounter. The initial fetal cardiology visit is seminal for families, [7,17,26] as it is when their child's diagnosis, prognosis, and potential management options are first discussed, and the expectations of care are established. [33] In this study, we explored the ways in which uncertainty is discussed: we aimed to answer the question of what aspects of uncertainty arise and how they are introduced during initial fetal cardiology consultations.

## 2 Methods

### 2.1 Design and setting

We conducted a single-center, observational, qualitative study of audio-recorded initial fetal cardiology consultations with families referred to fetal cardiologists for suspected cCHD in the summer of 2019. While this data collection was a part of a larger study, the data presented here was a primary focus. [34] Visits included clinicians and pregnant persons along with their partner or other support persons who accompanied them to clinic (hereafter referred to as families). In this study, cCHD included diagnoses with a moderate to high risk of affecting life expectancy [2,35] and a Society of Thoracic Surgeons – European Association for Cardio-Thoracic Surgery (STS-EACTS) mortality category ≥ 3 [36]. Non-native English speakers were included if they requested their counseling visit in English or if an in-person interpreter was present. Data on self-identified race, ethnicity, and family role were also collected.

### 2.2 Participant recruitment and data collection

All fetal cardiology physicians and nurse practitioners who perform prenatal consultation visits for cCHD within the participating clinic were eligible for study participation. Clinicians were invited to participate via email and the study was reviewed in detail in-person with each eligible clinician participant. Eligible families were consecutively invited to participate in the study in-person, immediately prior to their scheduled clinic visit. Visits included a fetal echocardiogram followed by counseling with a fetal cardiologist and fetal cardiology nurse practitioner. Audio-recordings were initiated after the fetal echocardiogram, at the start of counseling, with a digital voice recorder placed in the counseling room. Although many families would later meet with other subspecialists, such as a maternal fetal medicine obstetrician, neonatologist, social worker, geneticist, or palliative care physician, we only recorded the fetal cardiology visit. All participants (families and clinicians) gave written informed consent and verbal permission for audio-recording. This study was approved by the appropriate Institutional Review Boards. Additional details on institution and clinicians are omitted to protect confidentiality.

### 2.3 Data analysis

Audio-recordings were transcribed verbatim with personal identifiers removed. Content analysis, “a general term for identifying, organizing, and categorizing the content of narrative text,” per Patton (2014), [**37**] was carried out via an inductive and deductive approach. Given existing conceptions of illness uncertainty, we started with a deductive analysis using described causes of uncertainty, labeled in the literature as sources, meanings, or dimensions, and labeled them in our analysis as domains. Recognizing that there is no prior empirical work on causes and topics of uncertainty discussed in fetal cardiology, we were open to the nuances that would emerge from the recordings, which we included through inductive codes. [38] A preliminary codebook was created inductively by two coders (KWH and KS) based on the transcripts and deductively based on extant literature and clinical knowledge of the authors. A preliminary codebook was created inductively by two coders (KWH and KS) based on the transcripts and deductively based on extant literature and clinical knowledge of the authors. The authors include a medical psychologist (NAK), and physicians with specialties in general pediatrics (KS), palliative care (KWH, RMA, JM), pediatric cardiology (AK), and obstetrics-gynecology (JCC). The codebook was developed to code content (e.g., providing information, responding to emotion, discussing uncertainty). The codebook included codes with definitions, inclusion and exclusion criteria, and illustrative examples. [39] The same two coders (KWH and KS) then independently coded one to three transcripts at a time and met afterwards to systematically and iteratively refine the codebook, with mentorship by an expert qualitative researcher (JCC). JCC was also available to arbitrate if a conflict in interpretation and application of coding occurred. The codebook was revised through six rounds of revision. When no new codes or changes to the codebook were noted, the finalized codebook was applied to all transcripts. Coding discrepancies were adjudicated to agreement, and intercoder reliability was not calculated given method of adjudication. We used *NVivo12* (QSR International; Burlington, Massachusetts) to store and organize coding and memos regarding the coding process and decisions.

In this analysis, we focused on the codes and sub-codes related to uncertainty that were found in any portion of the recorded conversation. We included dialogue spoken by either clinicians or families. Uncertainty sub-codes were applied to any small phrase or full section of transcribed text related to various types of uncertainty. Different degrees of uncertainty were included. We then examined patterns and relationships among the codes, noting when uncertainty was raised, how, and by whom, and identifying content and topics associated with mentions of uncertainty. Quantitative data regarding the frequency of time spent discussing each uncertainty topic is included to provide context. To calculate the percentage of words spoken relating to uncertainty, we divided the number of words in a transcript with the codes of interest by the total number of words in that transcript; we then averaged those calculated proportions.

Our codebook incorporated conceptualizations from the literature on illness uncertainty, including from Han et al. (2011) and Mishel (1990), as described in the introduction. **[****18****,**[27], [28], [29], [30], [31], [32]**]** Uncertainty-related topics inevitably differ in terms of importance and degree of uncertainty based on an individual's perspective (i.e., parents may be uncertain about a topic that is certain to the clinician and vice versa). In our coding, we included uncertainty topics regardless of the degree or perspective.

## 3 Results

**Fig. 1:**
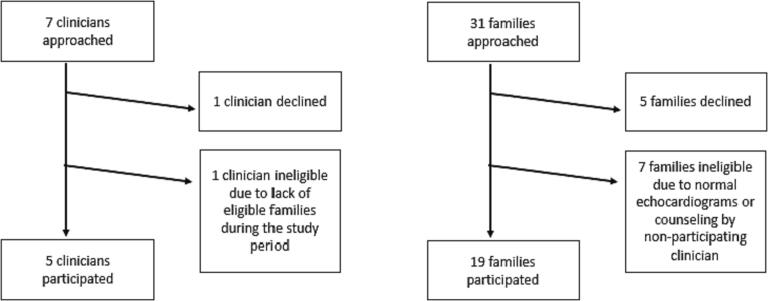
Recruitment flow diagram.

We approached 7 fetal cardiology clinicians (6 cardiologists, 1 nurse practitioner); 1 declined participation and 1 had no eligible patients during the study period in 2019. Five clinicians (4 cardiologists and 1 nurse practitioner) participated in the study. Of the 31 families approached for the study, 5 declined enrollment and 7 families were not eligible (Fig. 1), yielding an enrollment rate of 79% among eligible families (19/24). We obtained 19 audio-recorded conversations. Most pregnant persons identified as White and Non-Hispanic (74%), spoke English as their native language (79%), and had a partner or support person present (74%; Table 1). In these consultations, 13 different cardiac diagnoses were discussed, 7 with higher risk of mortality (Table 1). [35] Gestational age at time of consultation ranged from 20 to 35 weeks. The median counseling session duration was 37 min (IQR: 26–51 min). Each consultation consisted primarily of the clinician speaking, with only 11% of words spoken by families.

**Table 1:** Participant demographic characteristics.

	Characteristic	N	%
Clinicians (*n* = 5)	Time practicing in fetal cardiology, median (IQR), years	10 (9–11)	
Initial Fetal Cardiology Consultations (*n* = 19)	Duration of counseling, median (IQR), minutes	37 (26–51)	
	Words spoken by families, median (IQR), percent	11 (7–15)	
	Support person(s) present	14	74
	Other children present	3	16
	Language interpreter present	1	5
	Child named prior to consultation	10	53
Pregnant Persons (n = 19)	Gestation at first visit, median (IQR), weeks	26 (25–32)	
	Native language		
	English	15	79
	Spanish	1	5
	Arabic	1	5
	Kurdish	1	5
	Unspecified African Dialect	1	5
	Race/Ethnicity		
	White (European)	14	74
	White (Middle Eastern)	2	11
	Black / African American	2	11
	Hispanic / Latina	1	5
	Gravidity		
	1	3	16
	2	6	32
	≥ 3	10	53
	Parity		
	0	3	16
	1	6	32
	*≥* 2	10	53
	Prior miscarriages		
	0	14	74
	1	3	16
	2	2	11
	Family history of CHD	5	26
CHD Diagnoses (n = 19)	Lower risk of mortality †		
	Coarctation of the Aorta	2	11
	Tetralogy of Fallot	2	11
	Transposition of the Great Arteries (simple)	1	5
	Pulmonary Stenosis	2	11
	Atrioventricular Septal Defect	1	5
	Vessel aneurysm	2	11
	Higher risk of mortality†		
	Ebstein Anomaly (severe)	1	5
	Hypoplastic Left Heart Syndrome	2	11
	Hypoplastic Right Heart Syndrome	2	11
	Transposition of the Great Arteries (complex)	1	5
	Pulmonary Atresia	1	5
	Double outlet right ventricle and other anomalies	1	5
	Autoimmune complete heart block	1	5

Abbreviations: Congenital heart disease, CHD.

† Lower risk of mortality defined as score of ≤5; higher risk of mortality defined as score of >5 on the suggested CHD prognosis scale from Allan LD, Huggon IC. Counseling following a diagnosis of congenital heart disease. *Prenatal diagnosis*. 2004;24(13):1136–1142.

### 3.1 Topics of uncertainty discussed

When delivering and discussing the cardiac diagnosis, clinicians raised uncertainty in all 19 visits and within multiple topics of discussion. On average, 51% of words spoken during fetal cardiac consultations related to uncertainty. We identified seven topics of discussion in which uncertainty was mentioned: diagnosis, etiology, comorbidities, prognosis, childbirth, therapeutics, and logistics. Table 2 includes descriptions of each topic and Table 3 presents illustrative quotes. Of these, the most commonly discussed topics were diagnosis, prognosis, therapeutics, and logistics (all discussed in 19/19 encounters). Uncertainty topics most commonly raised by families were childbirth (9/19 encounters) and logistics during pregnancy and postpartum (11/19 encounters). Of the 19 encounters, 13 (68%) included discussions of uncertainty across all 7 topics, 5 (26%) included discussions across 6 topics, and 1 included discussion across 5 topics. The following sections provide detail on how uncertainty emerged within each of the seven topics, including illustrative quotations that are identified first by self-identified role (M = mother, F = father, C = clinician), then by study ID number (1–19).

**Table 2:** Descriptions of topics and domains of uncertainty.

Topics of Uncertainty		Present in Consultation
Diagnosis	Description of current and future medical condition	19/19
Etiology	How and why the medical condition is occurring	2/19 parents initiated 13/17 clinicians addressed
Co-morbidities	Additional medical conditions that may be diagnosed	17/19
Prognosis	Mortality risk and quality of life including functional outcome	19/19
Childbirth	Delivery of baby and events of immediate postpartum period	9/19 parents asked 18/19 clinicians discussed
Therapeutics	Medical management and surgical intervention	19/19
Logistics	Details regarding subsequent prenatal care and childcare specifics postpartum	11/19 parents initiated 19/19 clinicians discussed
Domains of Uncertainty		
Unpredictability	How future outcomes are indeterminate or unknown	19/19
Complexity	Complex or complicated features of information that may limit understanding	19/19
Lack of Information	Lack of reliable or adequate information	19/19

Note: In our coding, data was included under topics and domains regardless of the perspective or degree of uncertainty.

**Table 3:** Parental experiences of illness uncertainty during initial fetal cardiology consultation.

		Representative Questions		
Topics	Example Quotes	Unpredictability	Complexity	Lack of Information
Diagnosis	“I think I've got the important stuff, but we'll have to double-check everything I've told you when he gets here. You've had multiple people say that pump's too small. I think that's true. The details of it, could that be wrong? Yes” (C.1) “I wish I could tell you I've seen it change for the better, but that doesn't usually happen. I think this is going to stay the same. And… it could get worse.” (C.1) “We have to double-check not only what I've said about the heart, but what we've said about everything else about him too.” (C.13) “You're 32 weeks along. I'd like to try to look one more time before you deliver to see if we are starting to see any of the signs that could make us say yes, we need to be more concerned about a narrowing.” (C.15) “Knowing the heart is still developing, some things can change.” (C.19)	Will the diagnosis change during pregnancy or after the baby is born?	What are the various components of this diagnosis?	Where do I find more information about this diagnosis?
Etiology	“For a lot of them, we don't know exactly why it happens, and it's not the cause or fault of either parent or anything done in the pregnancy. It's just a difference that about one in a hundred babies have.” (C.4) The first thing parents always think is that “I must have done something wrong.” (C.7) “Your sister had a difference. So that increases your chance of having children that are different… We will probably do some genetic testing of your daughter in terms of the chance of this happening again, say, in her children… or future children you're planning on having.” (C.8) We know it's nothing you did” (C.11) “Thank you so much for explaining it. I think it took a little stress away. I was really stressed about it. I'm like, maybe it's the medication, maybe it's the smoking or maybe the stress I had from losing my sister. So, I was blaming myself for everything.” (M.17)	Will this happen again in a future pregnancy?	Why does my child have a heart difference?	Did I do something to cause this heart difference?
Co-morbidities	“30% of babies who have a difference in their heart, have a difference somewhere else. Sometimes the instructions are wrong for more than the heart. There are things called syndromes where it is multiple things different about your child… there is a difference in your heart and maybe a difference in your kidney or… in your hand. So, after birth, we have to check everything.” (C.7) “[DiGeorge syndrome] has a lot of potential issues with it from very, very minor to very, very severe…things that we see other than heart defects …They can have immunodeficiency problems, …problems regulating their calcium, …[and] varying degrees of learning disabilities.” (C.11) “We'll check his genes to see if there's any guide for special places we should check and then we're going to check him all over. We have one surprise. We want to make sure that there's no others.” (C.13) “Testing after birth is important because if she has a significant abnormality with her chromosomes that we don't think would be survivable in the long term, then we wouldn't recommend that she goes through the suffering of surgery. The same thing with the question about her brain, if the neonatologists, the baby experts after birth, feel like there's enough of an abnormality with her brain that they don't think she could survive long term. Then again, they don't want to put her through something hurtful from a heart standpoint if we don't think it would benefit her. This heart problem in and of itself, is repairable.” (C.16) “I'm going to talk about this as a heart only for now… but then we're going to double back because it's not a heart only problem” (C.20)	Will my baby have other serious medical conditions?	How might comorbidities impact my child's prognosis and care?	Who will counsel me more about the non-cardiac comorbidities?
Prognosis	“So, 80% are doing well, but 20% will have either died or gone on to a heart transplant from some of those problems, from irregular heartbeats or problems with the pump, or problems with a blood clot.” (C.1) “There's probably about a 50% chance this baby will survive to delivery, because of the severity of his heart lesion… even when we do everything we can from a medical and surgical perspective… there's probably about a 20% chance that this baby would survive past the neonatal period” (C.10) “So, sitting here in the womb right now, he does not care that he has Tetralogy of Fallot. He is getting what he needs, because, again, he has this extra blood vessel… this [diagnosis] is not something that will cause him to be sick or unwell. He should develop just fine. This is not a problem…within the womb.” (C.11) “You probably wouldn't notice my friends on the playground who have single heart pumps. If you rounded them all up and raced them, you'd pick mine out. They're not the fastest and they can't run as long, but they're out there doing their thing. Examples from some of my patients: one of my patients was a gymnast. He said, ‘I can do vault, but I can't do floor; that's too long.’ Another one said, ‘I need to take a break.’ Another one of mine was a dancer… and she said, ‘It's easy. I could dance for hours. You go a fast part, pose, breathe, little slow part, and then another fast part.’ They just need to rest more often.” (C.18). “The average IQ for children who have heart disease is the same as for everybody else. But more of my patients need extra help in school and have ADHD” (C.20).	Will my baby survive to birth? The newborn period?	If my baby does survive, what factors might impact their quality of life including cognitive function and physical abilities?	What do mortality risk percentages mean? How do they apply to my family?
Childbirth	“I was just going deliver wherever I was told… The only issue was, if we deliver here, I was curious if it would be an induction because I'm just worried that because my husband will be back at work by the time it's time for me to deliver. So, I'm worried about me going into labor at home and having to call him to get home from post, getting someone to come be with [our other children], and then it takes us an hour to get here” (M.4) “What that would look like is, again, clearly you are delivering here. You can deliver vaginally if that's the plan, that this would not change the method of delivery in any way, I anticipate that he will look just fine. You'll get a little bit of time to meet him.” (C.11) “If he looks perfectly fine, they're going to give him back to you to hold for about 10 min. It probably won't be longer than that because we have to start the medicine that keeps that blood vessel open.” (C.13) “I think we want you to deliver here just because we have the surgeons, we have the team who will put in pacemakers, but if you go into labor…we want you to go there and so that, then we can transfer you. … Because he has a heart block, this would be a C-section, I should have mentioned that. So, this will mean a C-section. So usually, we will plan for a C-section at 39 weeks, I'm sorry, to hopefully catch before he would go into labor, and he would try to come out on his own.” (C.14) “This doesn't mean that you have to have a c-section delivery. You can have a natural delivery. You can deliver on time; this doesn't mean you have to deliver early… This doesn't necessarily mean that you have to deliver here. Again, I'll talk with the folks at [redact] and we can help guide you if we think you should deliver here. If that's the case, if they would recommend that you deliver here, then they'll arrange to get you set up to see one of the high-risk obstetricians here.” (C.15)	When will I get to see or hold my baby after delivery?	Why would I need a C-section versus vaginal delivery?	Why do I need to deliver at a certain hospital, so far from my home?
Therapeutics	“So, we've talked about transplant and we've talked Norwood or Fontan or single ventricle combination. All those doctor's names. Those are the choices of things that we can offer [redact], and we will figure out the best [one]. There are some families who say, ‘Well, neither of these sounds very good to me.’ Without those, we would lose him. Okay. That actually is a choice some families make.” (C.1) “I think that there are definitely, we would have some options to fix this anatomically. I think when we have babies that have hearts that are so complex, final surgical plans cannot be made until after birth, when we can get echos directly on the baby and get a little bit more detailed because everything I get right now, I have to get through you to get to this baby. But the first big decision we have to make is do we feel like we can correct this heart where we can use both ventricles?” (C.6) “Lots of options that we have, but with the complexity of the heart and as early as we are, I cannot tell you which way that we would go.” (C.10) “In looking at your baby right now in measuring the pulmonary valve and the pulmonary arteries, I think it is likely that he would need that ductus to have enough oxygen going to his lungs, but I'm not a hundred percent sure. You are only 20 weeks. There are some babies with Tetralogy of Fallot that, that ductus will close, and they still have enough blood flow going into their lungs where we don't have to do something in the neonatal period, okay? So that's the first question. And I think I definitely am going to see you again through pregnancy. But ultimately the proof is after he gets here and doing echo pictures directly on him and sort of seeing how he's doing, about whether or not he's going to need an intervention before you guys go home from the hospital.” (C.11) “If you do get a heart [transplant], you have a two-chambered heart but you are on medications all of your life to keep your body from damaging it. It fights it off like a tumor. It's something called rejection. So that is also a lifelong problem. The mean time, median time, which means if you look at all the babies who get heart transplants, is the heart transplant lasts right now is about 22 years, which means some last even longer, some don't last as long. Some children have problems with rejection damaging their heart even as small children. If that happens, we try and re-transplant it. So, it is not a perfect pathway either… I think this pathway [the series of three surgeries] will work for him, but we are just going to have to double-check those coronary arteries. So that will be part of that first week too.” (C.13)	Will my baby need a heart transplant in the future? What additional interventions beyond heart surgery will my baby need?	What information will determine what kind of operation will my baby need? How will additional medications or a feeding tube impact how my baby recovers?	Who decides which interventions, if any, are pursued? What is the difference in risks and recoveries between interventions, such as a catheterization versus surgery?
Logistics	“So, we should expect to be back here several more times?” (M.3) “Then we'll likely be seeing you throughout pregnancy?… I'd rather not be bounced around.” (M.11) “We know we're going to have to be here for an extended time… we couldn't afford to stay in a hotel for two weeks or longer.” (M.20) “Some families do choose to relocate.” (C.21) “I'm going to be honest. I don't know [what other specialists I see today]. … I've got all of it until 1, just going appointment after appointment. I probably need to figure that one out.” (M.21)	How will my family handle caring for a baby with a life-threatening condition?	What are the roles of all the clinicians who are or will be involved in our care?	Who should I expect to coordinate prenatal care?

#### 3.1.1 Diagnosis

On arrival, some families were familiar with the reason for fetal cardiology consultation while others (6/19) were not. Families communicated unfamiliarity or lack of understanding of the reason for consultation. As one parent explained to the cardiologist, “They just say they see something. It wasn't normal” (M.15). During explanations and descriptions of the fetal cardiac diagnosis, clinicians would mention uncertainty pertaining to the diagnosis and unpredictability of how the condition may evolve or change as the pregnancy progresses; “Knowing the heart is still developing, some things can change” (C.19) and “I'm not sure we will know [the details of the diagnosis] for sure until after he's born” (C.15).

#### 3.1.2 Etiology

In two encounters, parents specifically reflected on the uncertainty of whether their actions had caused the diagnosis; in most other visits (13/17), clinicians explicitly addressed an assumed parental uncertainty about what caused the heart anomaly. One clinician said, “The first thing parents always think is, ‘Did I do something to cause this?’… The instructions for the heart were not right. You did not cause this” (C.13). Others shared, “This is not your fault” (C.6) and “Sometimes stuff just happens, you don't know why” (C.2).

#### 3.1.3 Comorbidities

In most encounters (17/19), clinicians discussed comorbidities, both those identified already and the evaluation for other comorbidities in the future. Uncertainty was raised as clinicians warned that the medical condition could include more than the heart anomaly. As one clinician counseled, “30% of babies who have differences of their heart have differences of other organs” (C.5). Cardiologists also raised uncertainty as cCHD may be part of a syndrome: “We'll check his genes… and then we're going to check him all over. We have one surprise. We want to make sure that there's no others” (C.13).

#### 3.1.4 Prognosis

In every encounter, clinicians discussed prognostic uncertainty, including anticipated symptoms, morbidity and mortality risks, potential complications, and future quality of life. One clinician said, “[Surgical intervention] gives a lot of my patients [a] pretty good life, but it has a really hard beginning and there's no promises they can [survive]” (C.1). In most encounters (13/19), clinicians specifically discussed mortality risk in relation to prognosis. Typically, mortality statistics were offered, which inherently communicated a degree of uncertainty. As in the following exchange, clinicians discussed this uncertainty with families:

Pregnant Mother: “Does a baby like this survive after all this?” (M.6)

Clinician: “Your baby's chance of survival is between 10 and 25%… which means yes, on occasion we had babies with this survive.” (C.6)

Clinicians also discussed uncertain prognosis regarding other outcomes, such as neurodevelopmental, physical, and mental health outcomes and quality of life. For example, clinicians raised the potential for developmental delays; “[It is] more common for them to have delayed milestones, [to] walk or talk late,” (C.18) and “[They have] a higher chance…of having attention deficit disorder or needing help at school” (C.13).

#### 3.1.5 Childbirth

In nearly every encounter (18/19), clinicians discussed the uncertainties surrounding the details of childbirth, including its timing, location, and method of delivery. In many encounters (9/19), families raised concerns regarding childbirth. As one mother explained to the clinician, “My major questions were timeframes and what to expect at birth” (M.4). Parents asked about timing: “Do you suspect her coming early or on time?” (M.8), about method of delivery, “This [diagnosis] has nothing to do with how I deliver?” (M.2), and about what will happen in the delivery room, “You will take [the baby] away right after birth?” (F.7).

In response to these family concerns, most clinicians discussed the inherent uncertainty. They often explained that the evolving cardiac condition in utero may have implications for aspects of planning for childbirth. As one clinician explained, “The delivery route goes differently if this [hole between the atria] is to get smaller” (C.18). Another stated, “We'll be watching…the measurements… because if that changes, it changes things about our delivery plan” (C.1). And another cautioned, “When she's born in the delivery room, she probably will not get to lay on your chest…It all depends on how she's doing.” (C.20).

#### 3.1.6 Therapeutics

During every consultation, clinicians discussed uncertainties regarding medical and surgical therapeutics that could be indicated based on the child's clinical status, such as intubation, prostaglandin, or artificial nutrition. For some cardiac diagnoses, discussion included uncertainty about whether surgery would be necessary immediately after birth; “Ultimately, the proof is after [the baby] gets here… whether or not he's going to need [surgical] intervention before you guys go home from the hospital” (C.11) and “This is going to be a day-by-day, play-by-play thing about how she is and what options we have” (C.10). For single-ventricle diagnoses, clinicians explained, the “series of three operations… sometimes that's not the best route and sometimes we have to do a heart transplant” (C.1). In a minority of encounters, clinicians acknowledged the choice for no surgical intervention; “There are some families who say, ‘Well, neither of these [heart surgeries or transplant] sounds very good to me.’ Without those, we would lose him. Okay. That… is a choice some families make” (C.1).

#### 3.1.7 Logistics

In every visit, uncertainty was raised regarding logistical concerns, such as the details of subsequent prenatal care visits or the specifics of caring for a child with cCHD; in many encounters (11/19), families initiated these discussions. For example, when asked whether she was scheduled to see the high-risk obstetrician later in the day, one mother responded, “I'm going to be honest. I don't know… I've got… appointment after appointment. I probably need to figure that one out” (M.21). Other mothers asked, “So, we should expect to be back here several more times?” (M.3), and “Then we'll likely be seeing you throughout pregnancy?… I'd rather not be bounced around” (M.11).

Another logistical uncertainty families faced was how they would manage caring for a child with a life-threatening condition, immediately after birth, during prolonged hospitalization, and after surgical intervention. As one mother explained, “We know we're going to have to be here for an extended time…[and] we couldn't afford to stay in a hotel for two weeks or longer” (M.20). Some clinicians introduced housing options; one acknowledged that “some families do choose to relocate” (C.21) and face the logistical hurdles and stress of moving closer to the hospital and away from their support system.

### 3.2 Illness uncertainty domains

**Fig. 2:**
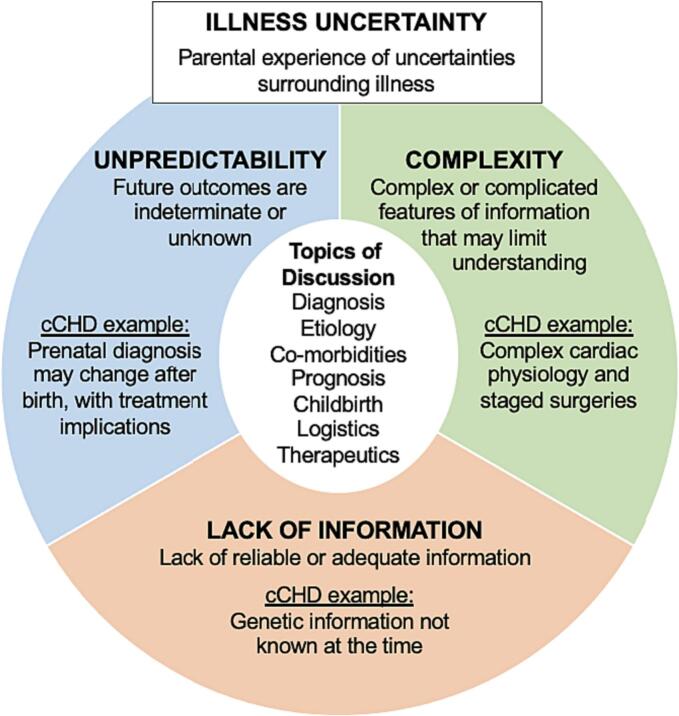
Conceptual model including three domains of illness uncertainty experienced by parents in the context of prenatal diagnosis of complex congenital heart disease (cCHD). Note: Color not needed for figure in print.

The three domains of illness uncertainty we identified – unpredictability, complexity, and lack of information – were represented across all seven topics of uncertainty discussed in initial fetal cardiology consultations. In other words, those domains were “manifest in a variety of concrete, substantive [topics],” as Han et al. (2011) described in his conceptualization. [27] Fig. 2, directly informed by work of Han et al. (2011) and Mishel (1983) on domains of uncertainty in healthcare, [27,32] depicts how the identified topics fit with the illness uncertainty domains. Table 2 includes descriptions of illness uncertainty domains and topics. Table 3 includes exemplar quotes and representative questions asked by parents and/or addressed by clinicians in our data.

#### 3.2.1 Unpredictability

Among the three illness uncertainty domains, unpredictability elicited anticipation and disclosure of psychological distress. As one clinician reflected, “the hardest thing is [taking it] one day at a time” (C.5). A mother echoed the difficulty of facing unpredictability: “I think that's our big thing right now is just, you don't know what, how severe or mild this is going to be until it's here… so we're just kind of waiting” (M.19).

#### 3.2.2 Complexity

Clinicians acknowledged complexity and responded by providing information to guide and prepare families. One clinician highlighted the complexity of medical terms used; “Whether we use the term, ‘double outlet right ventricle’ or not… I just want you to hear that term. It's more than an anatomy term. It is important for the surgeon after birth in the way that he approaches things” (C.5). Another warned of additional uncertainty that may arise over time; “There will be [more] information coming… that often brings up more questions” (C.1).

#### 3.2.3 Lack of information

Clinicians recognized how lack of information affected the prenatal diagnostic process. Many explicitly acknowledged the limitations of the tools for diagnosis and prognostication during pregnancy. For example, one clinician said, “I could have missed something” (C.7).

## 4 Discussion and conclusion

### 4.1 Discussion

This study examined clinician-family conversations during initial fetal cardiology consultations to describe what topics of uncertainty are discussed. We found that uncertainty was raised in all consultations and featured in half of all dialogue. Clinicians raised most topics and focused on diagnostic, prognostic, and therapeutic uncertainty. Families contributed a minority of the dialogue.

Our findings highlight the prominence of uncertainty in discussions with families at the time of prenatal cardiac diagnosis. Prior literature has recommended that clinicians across medical specialties explicitly discuss uncertainties with patients to facilitate shared decision-making. [40,41] Families receiving a prenatal cardiac diagnosis face present-time decisions about whether to pursue further prenatal testing and whether to continue with the pregnancy. [42] They also face decisions about the future, such as where to deliver their baby and whether to pursue interventions after birth. In these conversations about current and future decisions, clinicians in our study explicitly discussed what was uncertain and acknowledged the limits of what could be known in the prenatal setting. Interestingly, parental uncertainties relating to whether to continue the pregnancy were not found in our study. This may have been due to local legal restrictions (i.e., very few pregnant individuals were less than the gestational age of viability when elective termination may have been an in-state option), lack of trust in healthcare providers, or feeling the cardiologist was not the right person with whom to talk to about this topic.

While both clinicians and families discussed uncertainty related to logistics, other topics of uncertainty raised differed; clinicians most commonly discussed uncertainty related to diagnosis, prognosis, and therapeutics while families raised uncertainty about childbirth. Which uncertainties to discuss and how to deliver information is often influenced by the purpose of the clinical encounter. [30] In an initial fetal cardiology consultation, clinicians and families may have different priorities for the visit. Fetal cardiology clinicians aim to provide an accurate working diagnosis and to counsel families on that diagnosis and its implications. [43,44] They also provide obstetricians with guidance on future monitoring, delivery planning, and additional subspecialty involvement. [45] Parents' specific goals for the initial versus subsequent fetal cardiology encounters are not known. Although parental experiences of receiving a prenatal cardiac diagnosis have been assessed and show they often express a need for additional written or web-based information and guidance on how to access psychological and other forms of support, [6,46] the best way to operationalize that information specifically in the initial fetal cardiology encounter is not known.

Families in this study spoke a minority of words (11% of total words spoken), but the topics they raised may give us signal as to what areas of uncertainty are most salient for families. The limited dialogue from families suggests that parents may be too overwhelmed, shocked, or distressed at time of diagnosis to formulate specific questions, [6] but the clinical uncertainties they do raise, primarily focused on childbirth and logistics, are critically important to their pregnancy experience and identity as parents. Previous studies have highlighted the practical uncertainties families experience, [30,47] such as concerns about social support (e.g., what support will I have from my family or from my workplace?) and financial challenges (e.g., what costs will my insurance cover?). To address the varied uncertainties families face, fetal cardiologists could acknowledge those outside of their area of expertise [40] and identify when and by whom those uncertainties would be addressed by other members of the multidisciplinary team, including nurse coordinators, psychologists, and social workers. [48]

#### 4.1.1 Study limitations

Our study findings should be interpreted within the context of a few key limitations. This study was limited to one academic institution and included only five clinicians. While clinicians reported a wide range of prior clinical experience and consultations included many different cardiac diagnoses, the structure and focus of conversations may differ across a larger sample or in other geographical regions and cultures. Additional variables, such as time elapsed since referral, language barriers, gestational age, and clinician years of experience, likely influenced the uncertainty discussed and should be investigated in future larger studies. While families' perceptions of uncertainty were not directly assessed, clinician-family discussions of uncertainties undoubtably contribute to parents' experiences of illness uncertainty. Based on the goal of this study, only dialogue from initial visits were included; dialogue in subsequent prenatal visits may differ. Additionally, while the Hawthorne effect is a potential limitation, [49] the authors did not note any obvious deviation from initial fetal cardiology conversations outside of this sample.

### 4.2 Innovation

Despite these limitations, our study design and results are conceptually and methodologically innovative. Conceptually, this work highlighted our previous limited knowledge of the central role of uncertainty – including medical, social, and psychological uncertainties for family, clinician, and fetal patient – at the time of a prenatal cCHD diagnosis. As a result, we utilized multiple conceptualizations of uncertainty within our coding scheme to adequately capture its many facets in the prenatal period. Methodologically, this study is innovative as one of the first to examine audio-recorded dialogue between fetal cardiology clinicians and families in this context. [50] While in some other areas of medicine, clinicians struggle to name or address uncertainty, [51,52] this innovative methodology allowed us to quantify that when diagnosing cCHD prenatally, fetal cardiology clinicians focus at least half of the initial encounter on uncertainties related to that diagnosis.

Our study results not only highlight that fetal cardiology clinicians focus half of the initial encounter on uncertainties, but also identify the foci of uncertainty discussed in these consultations; however, whether those foci are desired by and beneficial to families is unknown. While parents vary in their individual emotional responses to a diagnosis of serious illness, [53] those who encounter more uncertainty tend to report greater psychological distress. [[54], [55], [56], [57], [58]] That being said, the uncertainty itself is not inherently negative; parents may need assistance developing increased tolerance of uncertainty, clinicians may need to improve their communication, and enhanced multidisciplinary support may need to be provided. As demonstrated in our study, clinician communication about uncertainty varies widely which may result from the lack of formal education on counseling techniques for most fetal cardiologists. [59] Communication skills training for fetal cardiology clinicians may support conversations about uncertainty and improve patient care. [6,46,59]

Subsequent work will first need to explore how families and clinicians experience uncertainty at the time of prenatal cCHD diagnosis and how they perceive optimal communication about uncertainties surrounding illness in that context. For example, clinicians may assume all families respond to uncertainty by seeking additional information; however, parents may differ in their informational needs and preferences and may not, for example, want to receive all information at once. [60] Innovative methodologies, such as concept mapping, may ideally assess a wide array of perspectives to include those previously lacking in the literature, such as fathers and bereaved parents, and individuals from groups historically excluded from research, including racial-ethnic minorities and lower socioeconomic groups. Such investigation will generate empirical recommendations for innovatively improving communication about uncertainties in the prenatal cardiology visit. Additionally, a framework for discussing uncertainties to optimize family outcomes, such as psychological adjustment and quality of life, will need to be developed. Developing a framework for discussing uncertainties will include re-envisioning the objectives of a prenatal cardiology consultation, training clinicians in communication, and providing families with the resources to address, mitigate, or embrace uncertainty as appropriate.

### 4.3 Conclusions

In initial fetal cardiology encounters, half the dialogue is focused on uncertainty. Given the known association between illness uncertainty and parental psychological distress, reexamining elements of the initial fetal cardiology consultation from family and clinician perspectives could help to prioritize content and optimize delivery of uncertainty-related topics. Approaches to support clinician-family communication about uncertainty could improve outcomes associated with fetal counseling and help parents and caregivers adjust to their child's diagnosis of critical or chronic illness.

## Funding

Research reported in this publication was supported by the 10.13039/100000102Health Resources and Services Administration (HRSA) under the National Research Service Award (NRSA) for Primary Care Research Award T32 HP22240 (KWH, KS), the 10.13039/100000050National Heart, Lung, And Blood Institute of the 10.13039/100000002National Institutes of Health under Award R38 HL143619 (KWH), and the 10.13039/100006108National Center for Advancing Translational Sciences of the 10.13039/100000002National Institutes of Health Award UL1 TR002243 (KWH). The content is solely the responsibility of the authors and does not necessarily represent the official views of the National Institutes of Health. Dr. Kasparian is the recipient of a 10.13039/501100001030National Heart Foundation of Australia Fellowship (101229) and support from the Heart Institute Research Core (HIRC) at Cincinnati Children's Hospital.

## CRediT authorship contribution statement

**Kelly W. Harris:** Conceptualization, Data curation, Formal analysis, Funding acquisition, Investigation, Methodology, Project administration, Resources, Software, Validation, Visualization, Writing – original draft, Writing – review & editing. **Kelsey Schweiberger:** Conceptualization, Data curation, Formal analysis, Funding acquisition, Methodology, Validation, Visualization, Writing – original draft, Writing – review & editing. **Ann Kavanaugh-McHugh:** Conceptualization, Validation, Writing – review & editing. **Robert M. Arnold:** Conceptualization, Validation, Writing – review & editing, Visualization. **Jessica Merlin:** Conceptualization, Validation, Visualization, Writing – review & editing. **Judy C. Chang:** Conceptualization, Formal analysis, Methodology, Supervision, Validation, Visualization, Writing – original draft, Writing – review & editing. **Nadine A. Kasparian:** Conceptualization, Formal analysis, Methodology, Supervision, Validation, Visualization, Writing – original draft, Writing – review & editing.

## Declaration of competing interest

There are no competing interests that relate to any authors to report.

## Data Availability

De-identified data that support the findings of this study are available on request from the corresponding author; data-use agreements will need to be completed and approved to share data. The data are not publicly available due to privacy and ethical restrictions.
